# Childhood Abuse and Intimate Partner Violence Victimization Among Filipina and South Asian Women in the United States

**DOI:** 10.1089/whr.2019.0001

**Published:** 2020-01-20

**Authors:** Akiko Kamimura, Maziar M. Nourian, Nushean Assasnik, Kimiya Nourian, Kathy Franchek-Roa

**Affiliations:** ^1^ Department of Sociology, University of Utah, Salt Lake City, Utah.; ^2^ Department of Anesthesiology, Vanderbilt University Medical Center, Nashville, Tennessee.; ^3^ School of Medicine, University of Utah, Salt Lake City, Utah.

**Keywords:** childhood abuse, Filipina, immigrant women, intimate partner violence, South Asian

## Abstract

***Background:*** Intimate partner violence (IPV) is a common form of interpersonal violence and impacts the health and well-being of victims over their lifetime. Many victims of IPV experience multiple types of victimization throughout their lives, often starting in childhood. The prevalence of IPV victimization of women varies among different race/ethnic groups. The purpose of this project is to examine childhood abuse among Filipina and South Asian women living in the United States who had experienced IPV.

***Methods:*** Data were extracted from Lifecourse Experiences of Intimate Partner Violence and Help-Seeking among Filipina, Indian, and Pakistani Women: Implications for Justice System Responses 2007–2009 (San Francisco, CA) (ICPSR 29682). Data were collected from 143 women (87 Filipina and 56 South Asian (*i.e.*, Indian or Pakistani) aged between 18 and 60 years who had been a victim of IPV and lived in the United States.

***Results:*** Although both Filipina and South Asian women who had experienced IPV reported a high prevalence of childhood abuse, Filipina women reported a higher prevalence than South Asian women. South Asian women were more likely to have first experienced IPV at a younger age and sought some form of IPV services as compared with Filipina women. The factors associated with experiencing all the types of IPV victimization included younger age at the first physical IPV victimization experience and higher educational attainment.

***Conclusions:*** Future research should examine the cumulative victimization of childhood abuse and IPV among Asian populations and its impact on health.

## Introduction

Intimate partner violence (IPV) is a common form of interpersonal violence and impacts the health and well-being of victims.^1^ IPV refers to a pattern of coercive tactics that may include physical, sexual, and emotional abuse that are used by one partner against another to gain power and control over the partner and the relationship.^2^ Many victims of IPV experience multiple types of victimization throughout their lifespan that often start in childhood.^3–5^ Despite the high prevalence, many victims of IPV often do not seek formal help to leave an abusive relationship.^6^

Childhood physical and sexual abuse and childhood exposure to IPV were risk factors for perpetrating or being a victim of IPV.^7–9^ Moreover, as the number of childhood violent exposures increased, the risk of being a victim of IPV or perpetrating IPV increased.^9^ A study of young Thai adults found that childhood exposure to IPV and physical abuse was highly predictive of IPV victimization in adulthood.^10^ In addition, multiple forms of victimization across the lifespan are associated with poorer health in adulthood.^11–14^ This compelling evidence that violence during childhood is linked to violence during adulthood makes violence across the lifespan an important public health concern.

The prevalence of IPV victimization of women varies among different racial/ethnic groups. A U.S. survey conducted in 2011 found that the prevalence of physical IPV victimization among women varied by race/ethnicity, with Native American females having the highest prevalence (51.7%), followed by black non-Hispanic (41.2%), white non-Hispanic (30.5%), Hispanic (29.7%), and Asian females (15.3%).^15^

Although the results of this national survey suggest that Asian women have a lower prevalence of physical IPV victimization than other racial/ethnic groups living in the United States, several studies have found a much higher prevalence of physical IPV victimization among some of the Asian subgroups.^16^ For example, previous studies have found victimization prevalence rates among South Asian (Indian and Pakistani) women in the United States to be as high as 44% for physical IPV victimization and 79% for childhood abuse.^17,18^ In addition, >30% of female Filipino American college students report being physically abused by an intimate partner.^19^

There are also methodological challenges in identifying the prevalence of IPV because the comparison of prevalence rates for different groups can be affected by how abuse is measured.^20^ For example, variations in the types of violence being measured, subjective perceptions of violence versus objective violence actions, and the duration and frequency of the violence can make comparison difficult.^20^

In addition, there are racial/ethnic differences in subjective perceptions of IPV. Studies that have looked at the prevalence of justification or perceived societal tolerance for IPV showed a wide range of acceptance from <1% up to 86% depending on the country and the given scenario.^21–24^ A study that reviewed 23 articles representing 67 countries found that reasons to justify IPV included victim neglects child, poor home care, argues with husband, or refuses sex.^24^ Surprisingly in the EU (39%), Spain (54%), and the United States (23%), at least one in five justified IPV as being the victim's fault because the victim secretly wants to be abused.^24^

Likewise, the 2013 National Abuse Statistics shows that Asians had the lowest prevalence of child fatalities as a result of child abuse among racial/ethnic groups in the United States.^25^ However, some studies indicate that the prevalence of physical childhood abuse is higher among Asian American families than the U.S. general population.^26,27^ Some of the Asian subgroups including Indians and Filipinos have a higher prevalence of family violence than the average prevalence among all Asian subgroups.^28^ A 2006 study reported a prevalence of physical child abuse of 50.7% among Filipino families and 47.8% among Asian Indian families in the United States.^29^

The differences in the prevalence of childhood abuse and IPV victimization among Asian subgroups in the United States indicate that the profiles of childhood abuse and IPV need to be examined by their racial/ethnic subgroups. IPV in Asian immigrant communities is related to cultural, societal, and individual/family context.^30^ Yet, such context can vary across Asian subgroups and thus each Asian subgroup may have different experiences of childhood abuse and IPV. Furthermore, little is known about the association between childhood abuse and IPV during adulthood among Asian subgroups.

Since childhood abuse affects the health and well-being of a victim across the lifespan,^31^ focusing on this association of victimization during childhood and adulthood is important when assisting Asian women who are IPV victims. Among South Asian women, a study focused on Indians and Pakistanis, who are the two largest subgroups among South Asians in the United States with high prevalence of IPV victimizimation occurring in their coutries of origin, showed the lifetime prevalence of physical or sexual IPV is 37.9% in India and of physical IPV is 26% in the Philippines.^32,33^

### Filipinas and IPV in the United States

Approximately 17% of the Asian population in the United States are Filipinos,^34^ with 69% of those being foreign born.^35^ The average family income of Filipinos living in the United States is higher than the U.S. average.^36^ The population of Filipinos living in the United States is rapidly growing; however, this minority group is understudied.^37^ Characteristics of Filipina women living in the United States include a high percentage of labor force participation, higher educational attainment, and gender equalities than most other Asian female subgroups living in the United States.^37^

Little is known about whether the characteristics of all Filipinas in the United States are shared by those who experience IPV. Cultural aspects of Filipina women living in the United States who had experienced IPV victimization include the following factors: feelings of shame not only for themselves but also for their culture; a tendency to not be open to discussing family problems with others; do not consider their victimization to be “abuse” and often blame the reason for physical IPV toward them as having to do with relationship conflict, alcohol, or stress.^38,39^

### South Asians (Indians and Pakistanis) and IPV in the United States

Approximately 22.5% of the Asian population in the United States are Asian Indians or Pakistanis with >87% being foreign born.^34,35^ Asian Indians have a higher family income than the U.S. average, whereas Pakistanis have about the same family income as the U.S. average.^36^ Based on a study conducted in Massachusetts, 44% of South Asians know a woman who has been physically abused or injured by her partner.^18^ The prevalence of physical IPV victimization among Asian women living in the United States is 19.6%, whereas the overall IPV prevalence (physical, sexual, and/or stalking) in the United States is 35.6%.^40^ Many of these women face significant barriers in seeking help because of strict gender roles and patriarchal standards found in these Asian families.^41^

Asian Indians living in the United States who reported higher levels of enculturation and patriarchal gender role attitudes were more likely to justify IPV against women.^42^ In addition, South Asian women who are immigrants to the United States are at a higher risk of IPV-related injury than South Asian women born in the United States.^43^ U.S. immigration policy may further complicate immigrant women's access to resources due to restrictions on the ability to gain employment in this country, which serves to further isolate these women.^44^

### Purpose

The purpose of this project is to examine the co-occurrence of childhood abuse and IPV victimization among Filipina and South Asian women living in the United States. Despite the potential significance of experiencing all three types of IPV (physical, sexual, and stalking), little is known about the association of childhood abuse and IPV victimization with all three types (physical, sexual, and stalking) as adults. This study contributes to increased understanding of the variable experiences of IPV among Asian subgroups and increases knowledge to better serve Asian women who have experienced IPV. In addition, there are few comparative studies that examine differences in child abuse-related experiences among women in Asian subgroups.

Research questions include (1) what is the prevalence of childhood abuse victimization among Filipina and South Asian women who have been victims of IPV and (2) what factors affect experiencing all types of IPV (physical, sexual, and stalking)? The outcome was experiencing all three types of IPV because experiencing multiple types of IPV is potentially a significant factor that impacts the well-being of IPV victims.^45,46^ The logistic regression analysis, which included all three types of IPV as an outcome and childhood abuse as a predictor, focused on experiencing childhood abuse and experiencing all three types of IPV during adulthood. The hypothesis tested was that childhood abuse victimization increases odds of experiencing all types of IPV.

## Methods

### Participants

Data were drawn from Lifecourse Experiences of Intimate Partner Violence and Help-Seeking among Filipina, Indian, and Pakistani Women: Implications for Justice System Responses 2007–2009 (San Francisco, CA) (ICPSR 29682). Data were collected from 143 women (87 Filipina and 56 South Asian—Indian or Pakistani) aged between 18 and 60 years who had been a victim of IPV and lived in the United States. These Asian subgroups were selected by the original data collectors because although Filipino and South Asians are the second and third largest Asian subgroups in the United States, research on IPV and child abuse among the populations is lacking.^47^ The original data collectors combined Indians and Pakistanis because both Indian and Pakistani are South Asian and share similarities in culture and demographics.

### Procedure

The participants were recruited through a variety of outreach methods such as flyers or advertisements. Data were collected using face-to-face individual interviews from 2007 to 2009 in a large metropolitan area in the western United States. The original collectors of the data explain the details of the data collection procedures in their report.^47^ The original data collectors obtained Institutional Review Board (IRB) approval. This study received exempt status because it was a secondary analysis of an existing anonymized data set. For this study, the permission and access to the data were obtained from ICPSR, which manages all permissions and access for the data sets.

### Measures

#### Intimate partner violence

The original data collectors obtained life course data on physical IPV, sexual IPV, and stalking by asking whether a participant experienced certain types of IPV incidents in a given year from age 16 years to age at the time of the interview. The original data collectors reconstructed the information by dichotomizing whether a participant experienced certain types of IPV incidents sometime in their life, in the study level data that were used for this study.

Physical IPV was measured by whether a participant experienced one or more of the following incidents (yes or no in lifetime): “pushed, grabbed, or shoved you”; “hit, slapped, or punched you (without object)”; “kicked you”; “strangled or choked you”; and “used knife, gun, or other objects (*e.g.*, cars, baseball bat, and bleach/acid).” Sexual IPV was measured by whether a participant experienced one or more of the following incidents (yes or no in lifetime): “forced you to have sex against your will”; “attempted to force you to have sex against your will”; and “forced you to have sex with others.”

Stalking was measured by whether a participant experienced one or more of the following incidents (yes or no in lifetime): “followed, spied on, stood outside home/work, or had someone else do that”; and “made unwanted phone calls, text messages, left unwanted letters, emails, gifts or items, or had someone else do that.”

#### IPV help seeking

Help-seeking behavior is important in describing victims of IPV because although help seeking increases the possibility to leave an abusive relationship and rebuild life after leaving, victims of IPV, especially who are minorities, are less likely to seek help. Additional information regarding help seeking can help guide public health intervention related to IPV in these groups of women. Participants were asked whether they had done the following help-seeking behaviors for IPV victimization: calling the police, seeking legal assistance, and using domestic violence (DV) programs (shelter or nonshelter services). These items were selected by the original data collectors.

#### Childhood abuse victimization

Childhood abuse victimization was asked separately from IPV victimization and was measured by asking whether a respondent had the following experiences before the age of 18 years: (1) physical abuse (*i.e.*, “pushed, grabbed, slapped, or kicked”; “left marks, bruises, or injuries”; and “physically punished”); (2) sexual abuse (*i.e.*, “touched or fondled you sexually”; “attempted sex/intercourse”; and “actually forced sex/intercourse”); and (3) emotional abuse (*i.e.*, “emotionally or verbally abused” and “made afraid that you might be hurt.” The original data included the frequency of childhood abuse victimization: never, once or twice, sometimes, often, or very often. Owing to the small sample size, there was not enough in each category. Thus, this study used the dichotomized information (yes or no) for analysis.

#### Demographic information

The following age information was asked: age at the time of interview, when immigrated to the United States, at the first physical and/or sexual IPV incident, and/or stalking experience. “Age at the first physical and/or sexual IPV incident” was included because IPV is often a lifecourse and recurrent experience.^48^ In addition, general demographic information was included: why a participant came to the United States (*e.g.*, to join family), educational attainment (bachelor's degree or higher and years of schooling), employment status, marital status, English proficiency, and whether U.S. born.

### Data analysis

Data were analyzed using SPSS (version 22). Descriptive statistics were used to describe the distribution of the outcome and independent variables. Comparisons between the two groups, Filipinas and Indians/Pakistanis, were conducted using Pearson's chi square tests for categorical variables if each cell had more than five respondents and independent samples *t*-tests for continuous variables because this study aims at analyzing Asian subgroups.

Logistic regression analysis was conducted to predict experiences of all types of IPV (physical, sexual, and stalking) and to examine associations between experiencing all types of IPV and sociodemographic factors, age at first physical IPV, and childhood abuse. All types of childhood abuse were combined because the experiences of each type of childhood abuse often overlap with those of other types of childhood abuse. A reference variable (Filipina) was included to control the differences between Filipinas and South Asians.

## Results

Table 1 summarizes the variables and the sociodemographic characteristics of the participants (*N* = 143). Overall, the most common reason for coming to the United States was to join family (30%) and nearly 20% came to the United States as a spouse/fiancée. Overall, almost half of the participants had a bachelor's degree or higher. Most of the participants were employed, rated their English proficiency as “very well” or “well,” and were not born in the United States. The average number of children was 1.87 (standard deviation = 1.6). Differences between the two groups included South Asian women as compared with Filipina women had significantly higher educational attainment (based on whether a participant had a bachelor's degree or higher), were married, and younger at the time of the interview and when they immigrated to the United States. More Filipina women than the South Asian women were employed and born in the United States. It is worth noting that the average educational attainment between the two groups was not significantly different if based on years of schooling. This is because years of schooling among Filipina participants had a wider range with some highly educated participants compared with those among South Asian participants.

**Table 1.** Sociodemographic Characteristics of Participants

	*Total* (N = *143*)	*Filipina* (n = *87*)	*South Asian*^a^ (n = *56*)	p^b^	F
Frequency (%)					
How came to the United States (top three reasons)					
To join family	42 (29.4)	32 (36.8)	10 (17.9)		
As a spouse/fiancée	26 (18.2)	4 (4.6)	22 (39.3)		
Visitor	14 (9.8)	10 (11.5)	4 (7.1)		
Bachelor's degree or higher	69 (48.3)	36 (41.4)	33 (58.9)	<0.05	
Currently employed	92 (64.3)	64 (73.6)	28 (50.0)	<0.01	
Married	45 (31.5)	17 (19.5)	28 (50.0)	<0.01	
English proficiency—very well or well	121 (84.6)	76 (87.4)	45 (80.4)	NS	
U.S. born	41 (28.7)	31 (35.6)	10 (17.9)	<0.05	

Mean (SD)					
Age	38.24 (10.91)	40.71 (11.13)	34.41 (9.45)	<0.05	5.38
Age when came to the United States	22.35 (11.49)	23.38 (12.80)	21.09 (9.65)	<0.05	5.57
Years of schooling	15.36 (3.89)	15.09 (3.84)	15.79 (3.97)	NS	0.30
No. of children	1.87 (1.6)	2.21 (1.52)	1.36 (1.60)	NS	0.05

No. (%) or mean (SD).

^a^ Indian: *n* = 45; Pakistani: *n* = 11.

^b^ *p*-Value denotes significance from Pearson's chi square tests between categorical variables (for cell size = >5 only), and independent samples tests for continuous variables comparing Filipina and South Asian participants.

NS, not significant; SD, standard deviation.

Table 2 describes lifetime experiences of IPV victimization and childhood abuse of the participants. The breakdown of childhood experiences is included to describe details of their childhood abuse experiences. The most common form of IPV victimization experienced by both groups was physical IPV (95.8%), followed by stalking (70%) and sexual IPV (59.4%). Nearly half of the participants (46.9%) had experienced all of the types of IPV victimization.

**Table 2.** Lifetime Experiences of Intimate Partner Violence and Child Abuse

	*Total* (N = *143*)	*Filipina* (n = *87*)	*South Asian*^a^ (n = *56*)	p	F
Frequency (%)					
IPV prevalence					
Physical IPV	137 (95.8)	83 (95.4)	54 (96.4)	NS	
Sexual IPV	85 (59.4)	49 (56.3)	36 (64.3)	NS	
Stalking	101 (70.6)	59 (67.8)	42 (75.0)	NS	
IPV type					
Physical, sexual, and stalking	67 (46.9)	37 (42.5)	30 (53.6)		
Physical and stalking	32 (22.4)	20 (23.0)	12 (21.4)		
Physical only	23 (16.1)	16 (18.4)	7 (12.5)		
Physical and sexual	15 (10.5)	10 (11.5)	5 (8.9)		
Other	6 (4.2)	4 (4.6)	2 (3.6)		
IPV help seeking (multiple answers)					
Called police	76 (53.1)	45 (51.7)	31 (55.4)	NS	
Sought legal assistance	72 (50.3)	38 (43.7)	34 (60.7)	<0.05	
Used DV shelter	45 (31.5)	22 (25.3)	23 (41.1)	<0.05	
Used nonshelter DV program	59 (41.3)	27 (31.0)	32 (57.1)	<0.01	
Childhood abuse prevalence (any)	104 (72.7)	70 (80.5)	34 (60.7)	<0.01	
Childhood abuse type					
Physical and emotional	53 (37.1)	37 (42.5)	16 (28.6)		
Physical, sexual, and emotional	37 (25.9)	26 (29.9)	11 (19.6)		
Other	53 (37.1)	24 (27.6)	29 (51.8)		
Childhood abuse type (breakdown) (multiple answers)					
Emotionally or verbally abused	80 (55.9)	55 (63.2)	25 (44.6)	<0.05	
Made afraid that she might be hurt	70 (49.0)	48 (55.2)	22 (39.3)	<0.05	
Pushed, grabbed, slapped, or kicked	69 (48.3)	45 (51.7)	24 (42.9)	NS	
Left marks, bruises, or injuries	43 (30.1)	31 (35.6)	12 (21.4)	NS	
Touched or fondled sexually	40 (28.0)	29 (33.3)	11 (19.6)	NS	
Attempted sex/intercourse against will	19 (13.3)	13 (14.9)	6 (10.7)	NS	
Actually forced sex/intercourse	11 (7.7)	7 (8.0)	4 (7.1)	NS	

Mean (SD)					
Age at first physical IPV	25.28 (8.41)	26.17 (9.47)	23.93 (6.28)	<0.01	7.13
Age at first sexual IPV	25.65 (9.61)	27.55 (10.75)	23.00 (7.07)	<0.01	8.51
Age at first stalking	26.41 (9.12)	27.73 (10.57)	24.55 (6.21)	<0.01	9.11

No. (%) or mean (SD)

^a^ Indian: *n* = 45; Pakistani: *n* = 11.

DV, domestic violence; IPV, intimate partner violence; NS, not significant.

With regard to IPV help-seeking behaviors of participants, over half of the participants reported that they had called the police. More South Asian women than Filipina women had sought legal assistance and used domestic shelter and nonshelter programs. There was no difference in help-seeking behavior between U.S.-born participants and non-U.S.-born participants, except for the “used nonshelter DV program,” which non-U.S.-born participants were more likely to utilize than U.S.-born participants (not shown in the table). In addition, Filipina women tended to be older at the time of their first IPV victimization (both physical and sexual) and stalking incident than South Asian women.

Although the majority of the participants (*n* = 104, 72.7%) reported being victims of abuse during childhood, with as many as one in four reporting that they had experienced all three types of childhood abuse, there were differences between the two groups. The Filipina participants reported an overall higher prevalence of childhood abuse (80.5%) than the South Asian participants (60.7%). Specifically, Filipina women had a higher prevalence of emotional/verbal abuse and fear that they would be hurt than South Asian women.

Table 3 gives predictors of having experienced all types of IPV. Having a college degree or higher and younger age at the first physical IPV were associated with experiencing all types of IPV (*p* < 0.05).

**Table 3.** Predictors of Experiencing All Types of Intimate Partner Violence (Physical, Sexual, and Stalking)

	B	*SE*	*Wald*	*Significance*	*Odds ratio*
Age	−0.03	0.02	1.62	NS	0.97
Married	0.62	0.46	1.86	NS	1.86
Employed	−0.52	0.44	1.38	NS	0.60
College or higher	0.98	0.43	5.16	<0.05	2.65
No. of children	−0.07	0.14	0.23	NS	0.94
U.S. born	0.96	0.54	3.11	NS	2.60
Filipina	0.53	0.48	1.21	NS	1.70
English well or very well	−0.89	0.62	2.06	NS	0.41
Age at first physical IPV	−0.076	0.03	5.45	<0.05	0.93
Childhood abuse (constant)	−0.32	0.51	0.40	NS	0.73
	1.70	1.10	2.40	NS	5.47
Model fit					
−2 Log likelihood	159.62				
Chi square	30.24				
Significance	<0.01				

Logistic regression.

NS, not significant; SE, standard error.

## Discussion

This study compared childhood abuse and adult IPV victimization among Filipina and South Asian women living in the United States. Although the hypothesis that childhood abuse victimization increases odds of experiencing all types of IPV was not supported, this study has three important main findings. First, Filipina women reported a higher prevalence of childhood abuse than South Asian women. Second, South Asian women were more likely to have first experienced IPV at a younger age and sought some form of IPV services as compared with Filipina women. Third, the factors associated with experiencing all the types of IPV victimization included younger age at the first physical IPV victimization and higher educational attainment.

More than half of the Filipina and South Asian women reported being a victim of childhood abuse (Filipina 80.5%; South Asian 60.7%). The co-occurrence of multiple types of victimization was common. Many previous studies have found that women who had experienced IPV victimization tended to have been abused in their childhood.^49,50^ Since more than half of the Filipina women had been victims of emotional abuse, parent–child interactions, including how children are raised, in a Filipino family should be examined to clarify how childhood emotional abuse would affect other types of childhood abuse and adult IPV victimization.

The finding that South Asian women tended to be younger than Filipino women at their first experience of IPV might be related to the finding that more South Asian women, than Filipino women, came to the United States as a spouse or fiancée rather than as a visitor or to join family. In South Asia, arranged marriages are very common and this type of marriage seems to occur even with South Asians living in the United States.^51,52^ There is a possibility that this type of arrangement increases the risk of IPV victimization because younger age when married has been found to be associated with an increased risk for IPV.^53^

The lifelong implications of childhood abuse and IPV victimization are critical to understand because cumulative abuse victimization, in which a victim experiences abuse over time and/or multiple types of abuse, is associated with poor health outcomes across the lifespan.^12^ Although about half of the women sought some form of services, most Filipina women did not seek legal assistance. Studies have found that access to services can prevent further episodes of violence victimization and thereby improve the health and well-being of victims.^54,55^

Our finding that a higher educational attainment was a risk factor associated with being a victim of multiple forms of IPV is in contrast to past studies that generally find that lower educational attainment is a risk factor for IPV victimization.^56,57^ This finding may be related to a reporting issue and be specific to some of the Asian subgroups. For example, studies in India suggest that women who are more educated than their husbands are more likely to report IPV victimization.^58^ The finding that this study group was overall highly educated may have contributed to these conflicting results.

This study has some important limitations to consider. First, the sample size was small; therefore, the different racial/ethnic groups among the Filipinas and South Asian women who were U.S. born, raised in the United States, or immigrated to the United States as adults were not able to be distinguished in the analysis. In many Asian cultures, talking about IPV experiences brings shame to the family, so it may have been difficult to recruit participants due to these cultural factors.^38^ Future research should examine different categories of subgroups with reference to reasons for coming to the United States and marrying within the same ethnic group, because this may increase the risk that people from a country with high rates of IPV will be abused in their new country of residence.

Second, the sample is a convenience sample that is not necessarily representative of all Filipina or South Asian women in the United States, and is limited to IPV victims. The small convenience sample does not necessarily justify the comparison between the two groups, and the results from such comparison may produce misleading information. Third, although this study focused on Indians and Pakistanis who are the two largest subgroups among South Asians in the United States, it is not known whether all South Asian groups are the same in terms of IPV and child abuse experiences. Fourth, the data do not include the ethnicity/race of the perpetrators.

Fifth, the geographic location of the data collection could influence the findings. San Francisco, where the data were collected has a large Asian community and thus may be different, with regard to help seeking, the availability of help in first languages, and policing strategies, from other regions in the United States. Finally, since the two populations have been understudied with regard to IPV victimization, it was challenging to conduct a systematic literature review and thus the literature review on these populations had to rely on the availability of literature.

Despite the limitations, this study provides new insights into the co-occurrence of childhood abuse and adult IPV victimization among Filipina and South Asian women living in the United States. Of note, an important consideration is the cultural beliefs of what constitutes childhood abuse. For example, in a recent study involving nine countries, culture variation accounted for one-third of the between-person variance.^59^ However, since this study described occurrences of certain incidences (*e.g.*, “pushed, grabbed, slapped, or kicked,”) rather than asking whether the participants considered that they had experienced childhood abuse, the cultural influences may have been mitigated. This information is useful in developing effective services to victims of IPV in these populations.

## Conclusions

This study examined childhood abuse among Filipina and South Asian women living in the United States who had experienced IPV. In particular, this study went beyond descriptive results previously reported^47^ by focusing on the association between IPV in adulthood and childhood abuse. This study found that most Filipina and South Asian women who had been victims of IPV in adulthood had also been victims of abuse during childhood. There were also differences between the two groups with regard to the prevalence of child abuse and age at first IPV experience. The finding in this study that higher educational attainment was associated with being a victim of IPV is in conflict with other studies that generally find that education is a protective factor against victimization.

Although Asian populations tend to be understudied or aggregated due to small population size in the United States, it is important to consider diversity among Asian subgroups related to childhood abuse and adult IPV victimization. Understanding help-seeking behaviors can help tailor community outreach to include the factors that will increase victims' help-seeking behaviors. Future studies should further examine the association between the co-occurrnece of childhood abuse and adult IPV victimization among Asian subgroups to develop culturally competent interventions and prevention programs for each subgroup.

In particular, future research should examine the cumulative victimization of childhood abuse and IPV among Asian populations and its impacts on health. Finally, although this study did not find an association between childhood abuse and experiencing all types of IPV, further research is necessary to identify other predictors and/or confounding factors that were not included in this study (*e.g.*, substance use, pregnancy, and criminal behavior) that can also affect the relationship between the two experiences.

## Abbreviations Used

DV: domestic violence
IPV: intimate partner violence
IRB: Institutional Review Board
SD: standard deviation
